# Lateral Curvature or Scoliosis in Children

**Published:** 1910-01-08

**Authors:** Alfred H. Tubby

**Affiliations:** Surgeon to Westminster Hospital


### HOSPITAL CLINICS.

### LATERAL CURVATURE OR SCOLIOSIS IN CHILDREN.

By ALFRED H. TUBBY, M.S., F.R.C.S.; Surgeon to Westminster Hospital.

(A Lecture at the Polyclinic on Monday, November 8, 1909.)

GENTLEMEN,—When I lectured here last time it was upon the subject of congenital club-foot, and I think that to-day I cannot do better than touch upon that difficult subject, lateral curvature or scoliosis as it affects children. By scoliosis is meant a deviation of the spine from the middle line, together with a rotation of the body. True scoliosis cannot exist without rotation.

I must first go into a few questions such as the ætiology, and the types of lateral curvature which are met with. This condition is essentially an affection of young life. It is comparatively rare before the age of five years; it becomes increasingly frequent between five and ten, and from ten to fifteen its appearance becomes more marked. I think we shall be correct in saying that its onset is best marked between eight and fourteen. And, unfortunately, it is much more common in the female sex than in the male, which is due, I think, to the fact that, in the first place, the muscular system in the female is not so well developed as in the male, and, in the second place, to the greater strain of puberty in that sex.

The varieties of lateral curvature clinically are the following: (1) There is a long "C" curve, the curve of the spine extending almost the whole distance, with a very slight and ill-defined return of the curve to the opposite side in the lower part of the back. It is generally to the left, and many appearances commence with this curve; it is impossible for such a curve to exist without some attempt at a compensatory curve to enable the patient to maintain his or her equilibrium. The long "C" curve form is also known as total scoliosis, the word "total" implying that it affects the whole or the greater part of the spine. It is the kind which you see in young weak-backed girls who suffer from mal-position at school and muscular fatigue. If the long curve "C" persists, it alters into the next type, the typical plain "S" curve, pointing in one direction in the dorsal region and in the opposite direction in the lumbar region. The commonest clinical type is two equal curves, right dorsal and left lumbar. In the third type you see three or more curves present, and this multiplication of curves may be so marked that I have seen recently a back in which there were five distinct curves, and another back in which six could be counted. Of course, the greater the number of curves the less must be the extent of each and the smaller its radius; and also the less will be the distortion of the body caused by them. There are other clinical types seen, which I must indicate by diagrams. One particularly interesting type is lateral curvature with posterior projection of the spine. In some patients' backs you will find when you are dealing with a double curve that at the meeting point of the two curves, or at the point where they cross over the middle line, there is a projection of two or three spinous processes. The significance of that is considerable, and it introduces an element of difficulty in diagnosis. The one symptom of spinal caries which is patent to everybody is the posterior projection of the spinous processes. The one symptom of lateral curvature is deviation of the spine from the middle line. In some cases of Potts' disease there may be a deviation of the spine from the middle line, and in some cases of scoliosis there may be a projection of the spines themselves. But the diagnostic difficulty is not very great in these cases, because you will find that when you direct the patients to bend forward and twist their backs the projecting spinous processes are, as a rule, freely movable; that is to say, there is none of that osseous ankylosis present in scoliosis which there is in Potts' disease, unless the scoliosis has existed for a long time and the vertebræ have become ankylosed together. Pain is very slight, as a rule, and there are other diagnostic signs. But I draw your attention to this point so that you may not think that every case of posterior projection of the spinous processes is necessarily Potts' disease, or every case of later deviation of vertebræ is necessarily scoliosis.

Lastly, there are those cases where there is a reversal to the normal curve to some extent. By reversal I mean that the normal dorsal region should project posteriorly, and the lumbar region anteriorly. The normal position of the dorsal region is one of kyphosis, and that of the lumbar region is one of lordosis. In some cases there is a distinct flattening of the dorsal region, combined below with a flattening of the lumbar region, and even perhaps a slight projection, showing that the normal curves have been reversed. When that condition occurs it indicates extreme rotation of the vertebræ. With regard to that question of rotation, I would mention one cause to you. One is often asked, How do you estimate the amount of rotation in a given patient? The usual answer is by the deviation of the spinous processes from the middle line. But that estimation, as I shall show you, is very fallacious. There is only one means of estimating the actual amount of rotation, and that is by noting the projection of the ribs on one side in the dorsal region and the projection of the loin on the other side in the lumbar region. To do that, tell the patient to cross the arms over the chest and bend down; and as he bends down the posterior aspects of the ribs become uncovered, and you can estimate at once the degree of displacement of the ribs and, therefore, the degree of rotation. I show you drawings to illustrate that. In the anterior view you will see that the lumbar vertebræ are enormously twisted round, some of them nearly through a right angle, and yet there is very little deviation of the spinous processes. And here comes a further difficult point. In scoliosis the vertebra undergoes two changes in position: lateral deviation, bodily carried over, say, to the right, and also twisted on itself, so that there is a body which has undergone change in two directions, first carrying outwards, and secondly the twisting round of the body to the convex side of the curve. You would probably say if the body were twisted to the convex side of the curve, the spinous processes should go to the other side. I grant that as far as rotation is concerned, but assuming the body is carried out some distance, the spines must be carried out some distance, and the spine follows the line of convexity of the curve.

I now pass to the consideration of the causes of scoliosis. It has recently been shown that congenital scoliosis, or scoliosis due to congenital causes, is more common than could possibly have been anticipated by examination by the ordinary methods; and the more frequently one has spines skiagraphed the more often do you find congenital changes. For instance, in this picture you find six instead of five lumbar vertebræ. The congenital changes which may exist in the spine are very much as follows. You may classify them as cases in which the normal number of vertebræ is exceeded, and cases in which the normal number is diminished. There may be as many as eight or nine cervical vertebræ, 13 or 14 dorsal vertebræ, and six lumbar vertebræ. But it is seldom that you find more than one region very much altered. I think it is correct to say that in some cases you may see as few as three cervical vertebræ; there is another case described in which only five cervical vertebræ were present, and other cases in which there may be only four. There may also be curious changes in the shape. The bodies of the vertebræ are, roughly, rhomboid in shape, and upon the perfection of this rhomboid shape depends to some extent the uprightness of the vertebral column. But imagine that it has developed a wedge, then all the vertebræ above will be inclined to fall off from the sloping upper edge of the wedge. That condition is very common, and accounts for many cases of scoliosis. It is not infrequent, in cases of cervical rib, to have a wedge-shaped vertebra interposed between the last cervical and the first dorsal vertebræ, and then the vertebræ above and below are twisted out of the right line. There are also other associated and well-marked abnormalities. The most common in the neck is the supernumerary or cervical rib, of which I have seen several examples, and often it is accompanied by an inclusion of the wedge-shaped vertebræ. There may be other curious changes; for instance, the body and the pedicles of the last lumbar vertebra are not united, so that the spine slips forward on the sacrum, and you have the curious condition known as spondylolisthesis. But I have said enough to show that congenital malformation of the vertebræ is a very potent factor in the production of scoliosis, and very important in prognosis, and it demands treatment along certain special lines. You must not regard a spine as hopeless from the point of view of treatment which is the subject of congenital malformation of the vertebræ.

The second group of causes of scoliosis is what may be called static causes. By "static" one means varied condition of standing and position; and these are frequently due to certain organic and intrinsic causes, which must be recognised and appreciated before they can be treated. One of the commonest of these is congenital asymmetry.

In a normal individual the lines drawn from 1 to 2 on either side will be equal, and lines drawn from 2 to 3 on either side will be equal; and you would not expect scoliosis to occur unless the muscular system was much weakened, or the patient got into very bad habits of standing. When these lines are asymmetrical you often find a curious condition of affairs. R 1 to R 3 may be greater than L 1 to L 3, or *vice versa*. If that be so you have to find out whether the want of symmetry exists in the two halves of the body, or in the two legs, or in both body and legs. In order to do that, the plan of measurement which I have indicated is very simple. You measure from the top of the sternal notch to the anterior superior spine, and from there to the internal malleoli. You may find that the two sides of the body are unequal, or the two legs are unequal, or that both are unequal. And then you have to apply appropriate corrections, both under the foot for standing and under the gluteal region in sitting. Many people are content to apply the correction beneath the foot; but they forget that scoliosis in the spine will, if the two sides of the body are unequal, exist as much in sitting as standing, and they forget to correct that sitting position. But the matter is not so simple as that. There is a curious added condition, which we indicate by the sign + or the sign —; and this brings us to the subject of crossed asymmetry. If one side is greater than the other, and we find that the right leg is smaller than the left, you can understand how, when the patient sits or stands, there must be a certain amount of deviation of the spine. But there is also another symptom: it is just in these cases of crossed asymmetry that you find that peculiar pelvic twist which is so difficult to remedy. And by pelvic twist I mean this: the line joining the two shoulders is not absolutely parallel to the line of the transverse diameter of the pelvis; but the transverse diameter of the pelvis makes, with the transverse diameter of the shoulders, an angle; and if the pelvis be twisted in this way, the spine must be twisted, and therefore scoliosis sets in. I might complicate it by telling you the difference between simple asymmetry and crossed asymmetry, but it would be rather too much to inflict on you. In crossed asymmetry you can understand how the patient's position varies in standing and in sitting, and how you must apply, in many cases of crossed asymmetry, correction beneath one side for standing and on the opposite side for sitting.

There are also a large number of other static causes which I may just run over briefly, such as shortening of one leg due to hip-joint disease, or to coxa vara; lengthening of one leg, due in some cases to coxa valga; shortening of one leg due to knee disease, and also the habit of standing on one foot which is frequently associated with flat-foot. You will meet with static causes in the upper extremities, such as paralysed upper extremity, or an upper extremity which has been partially removed. Take as an illustration the scoliosis which occurs in the condition known as wryneck. I also have an example which shows how scoliosis is produced in the infant by the habit of the nurse of carrying the child on the arm with the pelvis always inclined at an angle. So much for static causes. Now we come to constitutional scoliosis, and I use the word with some reluctance, because it scarcely expresses what I mean: I might call it "habitual." By that I mean curvature of the spine occurring in people whose muscular, bony, and ligamentous systems are not sufficiently strong to maintain them in the upright position under exercise and fatigue. These cases ultimately, as a rule, afford many of the worst examples of scoliosis. They are seen in girls between the ages of fourteen to eighteen who have long and narrow backs, with flexible, willowy spines; they are often anæmic, and unfortunately some of them have to work many hours a day to earn their living.

The result is that the spine begins to deviate, and what in the first case is fatigue becomes later an habitual position and then becomes fixed, and they then develop spinal curvature. In the first place, they have the long "C" curve which I have described to you. If the condition is not checked it changes over and becomes a double curve, a left dorsal and right lumbar. These are the cases which give surgeons a great deal of trouble and anxiety, and you cannot hope to treat them unless you recognise the general muscular insufficiency of the patient to meet the ordinary conditions of life. Under this head I might speak of the curvatures of school life. A large number of girls at about the age of fourteen develop curvature of the spine of varying extent. Some of those curvatures are due to bad positions in sitting and standing, whilst others are due to the fact that the muscular system is not sufficient to keep the spine upright during the fatigues incidental to school life, and especially those of writing and sitting in certain positions for a long time. I may, in that connection, allude to particular conditions which give rise to scoliosis in girls. One of them is playing the violin. I am convinced, from what I have seen, that, of all the pernicious positions in many girls, that assumed in playing the violin is the worst. The right shoulder is raised and the left shoulder is lowered and carried forward, with a curvature to the left in the dorsal region and a compensatory curve to the right in the lumbar region. I am constantly asked whether a patient may play the violin. If the girl has already a certain amount of curvature, even if it be very slight, the answer should be unhesitatingly "No." If it be already advanced, all practice on the violin must be stopped, or the patient will never get well. Another cause of a certain degree of curvature is playing hockey. There is a "hockey shoulder" in which the left shoulder is raised and carried forward, and a curvature is developed, also to the left in the dorsal region. These facts may sound far-fetched, but they are being recognised by the more intelligent mistresses and teachers, and in some schools hockey is taken out of the curriculum, and so is violin-playing. Another cause is the curious position in which children are taught to write. I have a very vivid recollection of my writing days. The pen was tied to my fingers by means of red tape, I was directed to lean over to the left side and point the head away. If there is any condition more likely to cause scoliosis it would be difficult to think of it. Inclining the pelvis to the left was a direct inducement to the formation of a large left dorsal curve, and in this way it elevated the right shoulder. I think the effect of teaching boys and girls to learn the sloping or Italian hand has been responsible for the production of much scoliosis. We call it the Italian hand, but they get it back on us by saying it is the English hand. The proper way is to make the pupil sit straight to the desk with both arms firmly on the desk, and teach them to write round-hand in that way.

Other causes of scoliosis are nerve causes. Some cases of nerve origin are very intractable, and others are very amenable to treatment. There is a curious form of curvature of the spine which occurs in connection with sciatica. The patient who is the subject of sciatica gets into the habit of flexing the leg to a slight extent, dropping the pelvis slightly on that side. The result is to develop lumbar curvature, almost always to the side on which the sciatica is. It is known as ischias scoliotica. That is only a temporary form of scoliosis. When the patient relaxes the strain he relaxes that position and the pelvis resumes its normal place and the lumbar curve disappears.

Then there is hysterical scoliosis. I have seen several cases of that. You are astounded at the extraordinary appearances of the patient. It occurs not only in young girls, one meets it from time to time in middle-aged men, and particularly after slight injury to the spine, and most particularly after a railway accident. The points about hysterical scoliosis are its comparatively sudden onset, the fact that it disappears entirely on suspension, and it does so also under an anæsthetic. And, despite extreme deviation of the spine, there is very little evidence of rotation of the ribs or evidence of rotation in the projection of one loin. You may further find out the nature of the case by watching the patient carefully and noting the further history. Several of my cases of this kind I have cured by the application of a good, strong current to one side or the other, and they have improved very quickly. Sometimes the threat of the battery is enough. The worst of the other cases of scoliosis, from a prognostic point of view, is that which is associated with infantile paralysis. If the muscles of one side of the back are entirely paralysed, the spine sags back to the paralysed side, and there is developed a curve with its convexity on the paralysed side. That may be called a most remarkable example of muscular asymmetry. Those cases in which there is weakness of muscles on one side, or destruction of erector spinæ muscles, are extremely difficult cases to treat, and cases which are adapted for the use of supports. You will find also lateral curvature associated with spastic paralysis, with Friedreich's disease or hereditary locomotor ataxy, and with rare conditions such as syringomyelia. I think there are half a dozen cases recorded of scoliosis occurring in the course of locomotor ataxy, but it is not true scoliosis in that sense; it is really an example of Charcot's disease occurring between one or two lumbar vertebræ and dislocation of the upper part off the lower part; and the upper dislocated part slides away to one side, while the lower part remains, more or less, in the middle line.

I now will briefly indicate the clinical types of curves which are met with. You may meet with cases which are purely lumbar. In the lumbar region there is a sharp curve, and a small return curve with the spine running up straight. These are usually in cases where there is shortness of one leg. The lumbar curvature may be one of two types: either the overhanging type or the non-overhanging type. The next is the lumbo-dorsal curve, where it begins a little higher up than the preceding and affects the lumbo-dorsal region, and is scarcely at all marked above that. There is also the semi-dorsal curve. Or you may have the ordinary dorso-lumbar curve, two curves which are roughly equal. Lastly, there may be cervical dorsal curves, which affect the lower part of the cervical region and the upper part of the dorsal region. The lumbar curves are met with especially in cases of inequality of the legs. The double dorso-lumbar curve is met with usually either as a constitutional condition, or as a result of a bad position in sitting or standing. The single dorsal curve is met with especially in rickets. The most common cause of spinal curvature in after life, and most certainly in children, is rickets. And you can understand how it is that rickety spines become curved. It is a physiological fact that the more a back is flexed the greater the amount of deviation from the middle line which the vertebræ can undergo; the more the back is held erect the less the deviation. In rickets the spine is characterised by the tendency and by the position which it assumes. The earliest sign of rickety spine is exaggerated flexion, and I have known flexion to be so exaggerated owing to the weakness of the muscles and the laxity of the parts that the child has been unable to sit upright. When the child has been put into the sitting position and you release it, the head droops forward until it nearly touches the knees. From what I have said you will readily see how it is that rickets bring about such serious conditions of lateral curvature. Beginning at the age of three or four, these curves often persist, and as the individual grows up the curvature becomes more marked, and I believe that the larger proportion of cases of severe curvature seen in adolescence and adult age are traceable to neglected rickety curves which commenced in early childhood.

